# Natural Convection within Inversed T-Shaped Enclosure Filled by Nano-Enhanced Phase Change Material: Numerical Investigation

**DOI:** 10.3390/nano12172917

**Published:** 2022-08-24

**Authors:** Aissa Abderrahmane, Mohammad Al-Khaleel, Abed Mourad, Houssem Laidoudi, Zied Driss, Obai Younis, Kamel Guedri, Riad Marzouki

**Affiliations:** 1 Laboratoire de Physique Quantique de la Matière et Modélisation Mathématique (LPQ3M), University Mustapha Stambouli of Mascara, Mascara 29000, Algeria; 2 Department of Mathematics, Khalifa University, Abu Dhabi 127788, United Arab Emirates; 3 Department of Mathematics, Yarmouk University, Irbid 21163, Jordan; 4 Faculty of Mechanical Engineering, University of Sciences and the Technology of Oran, Oran 31000, Algeria; 5 Laboratory of Electromechanical Systems (LASEM), National School of Engineers of Sfax (ENIS), University of Sfax (US), B.P. 1173, Road Soukra km 3.5, Sfax 3038, Tunisia; 6 Department of Mechanical Engineering, College of Engineering in Wadi Addwasir, Prince Sattam Bin Abdulaziz University, Wadi Addwasir 11911, Saudi Arabia; 7 Mechanical Engineering Department, College of Engineering and Islamic Architecture, Umm Al-Qura University, P.O. Box 5555, Makkah 21955, Saudi Arabia; 8 Chemistry Department, College of Science, King Khalid University, Abha 61413, Saudi Arabia; 9 Chemistry Department, Faculty of Sciences of Sfax, University of Sfax, Sfax 3038, Tunisia

**Keywords:** magnetohydrodynamics, inversed T-shaped enclosure, NEPCM, natural convection, nanofluid

## Abstract

Energy saving has always been a topic of great interest. The usage of nano-enhanced phase change material NePCM is one of the energy-saving methods that has gained increasing interest. In the current report, we intend to simulate the natural convection flow of NePCM inside an inverse T-shaped enclosure. The complex nature of the flow results from the following factors: the enclosure contains a hot trapezoidal fin on the bottom wall, the enclosure is saturated with pours media, and it is exposed to a magnetic field. The governing equations of the studied system are numerically addressed by the higher order Galerkin finite element method (GFEM). The impacts of the Darcy number (Da = 10^−2^–10^−5^), Rayleigh number (Ra = 10^3^–10^6^), nanoparticle volume fraction (φ = 0–0.08), and Hartmann number (Ha = 0–100) are analyzed. The results indicate that both local and average Nusselt numbers were considerably affected by Ra and Da values, while the influence of other parameters was negligible. Increasing Ra (increasing buoyancy force) from 10^3^ to 10^6^ enhanced the maximum average Nusselt number by 740%, while increasing Da (increasing the permeability) from 10^−5^ to 10^−2^ enhanced both the maximum average Nusselt number and the maximum local Nusselt number by the same rate (360%).

## 1. Introduction

In the last several decades, the heat transport of nanofluids in various shapes with varying outset and boundary conditions has been a popular research point. This is explained by the fact that these geometries have been widely used in real-world applications, such as building thermal management, electronic device cooling, biochemical and food processing, and renewable energy applications [1,2,3,4,5,6,7]. Raizah et al. [8] examined nanofluid natural convection (NC) flow inside a V-shaped cavity saturated with porous media. The findings showed that the best-case scenario for porous media is a horizontal heterogeneous porous medium. The buoyancy force is augmented with a Rayleigh number increase, which improves convective transport. In a triangular fin-shaped cavity, Khan et al. [9] presented a computational investigation of the convective heat transport of a hybrid nanofluid. Their findings demonstrate that both the nanoparticles’ Rayleigh number and solid volume percentage raise the local and average Nusselt numbers. In a uniquely formed cavity, Ghalambaz et al. [10] analyzed the heat transmission and irreversibility of a hybrid nanosuspension. The findings demonstrate that raising the nanoparticle concentration accelerates the rate of entropy formation for all Rayleigh number values. Asmadi et al. [11] investigated how a hybrid nanofluid transfers heat naturally by convection within a U-shaped container with varying heating configurations. The findings indicate that the continuous heating setting delivers the optimum heat removal performance, whereas the oscillating heating setting performed the poorest. The magnetohydrodynamic free convection flow and heat transfer in an angled U-shaped enclosure loaded with Cu-water nanofluid were studied by Nabwey et al. [12]. The findings demonstrate that the mean Nu increases with dimensionless heat source position but decreases with heat source length and Ha number. A numerical study on the free convection flow of a hybrid nanofluid in a reservoir with a trapezoidal form under the impact of partial magnetic fields was conducted by Geridonmez et al. [13]. According to the results, heat transmission and fluid movement are inhibited by the partial magnetic field’s vast effect zone.

Conventional heating and cooling systems rely on a working fluid with a limited thermal capacity. Several systems incorporate a greater flow or bigger volume to address this problem, which is an inadequate solution in some applications. In this context, NEPCM is one of the effective approaches for increasing the thermal efficiency of various systems. In this encapsulation technique, PCM is sealed inside nanoshells to prevent leakage. In this structure, the PCM-containing core layer may store and release enormous amounts of energy during melting and solidification at a constant fusion temperature. The use of NEPCM rather than only heat transfer fluid offers several benefits in many applications. NEPCM benefits from both heat transfer fluid and PCM characteristics [14,15,16,17]. For instance, it was recently shown that these could improve thermal storage characteristics and also for applications such as triple tube heat exchangers [18] or shell-and-tube heat exchangers [19]. To keep the temperature of lithium-ion batteries (LIBs) stable between 35 and 45 °C, Cao et al. [20] utilized water loaded with NEPCM particles. They found that raising the Reynolds number value from 70 to 100 increased the rate of heat transmission of LIBs by 12.1–17.2%. Raising the volume fraction from 0 to 3% also increased the heat transmission rate by 8.2–13.6%. Mohammadpour et al. [21] investigated NEPCM slurry’s hydrodynamic and heat transfer characteristics inside a microchannel heat sink featuring two circular synthetic jets. The thermal performance improvement is maximized at 28.5% at 0.2 nanoparticle volume fraction and 180 out-of-phase actuation, according to simulation findings. On the other hand, the figure of merit falls when the concentration of NEPCM rises. In a conical diffuser, Iachachene et al. [22] investigated the turbulent flow of Al_2_O_3_, NEPCM, and a mixture of the two. The NEPCM nanofluid had the lowest pressure drop and the largest heat transfer improvements inside the diffuser, according to the findings of this investigation. The Nusselt numbers of NEPCM/Al_2_O_3_ hybrid and Al_2_O_3_ nanofluids were enhanced by 10% and 6%, respectively, whereas the Nusselt number of NEPCM nanofluids were raised by 15%. Analytical research on the laminar flow and heat transmission of water jet impingement augmented with NEPCM slurry was conducted by Mohaghegh et al. [23]. The results indicate that NEPCM slurry may greatly increase the system’s cooling performance by increasing the liquid jet’s ability to store latent heat. However, an ideal NEPCM concentration results in the system’s maximal cooling performance (15%). To improve total heat transmission and lessen pressure drop, Doshi et al. [24] developed a water-based NEPCM nanofluid within a new microchannel with a wavy and irregular shape. The result demonstrates that the presence of NEPCM nanoparticles lowers the fluid domain temperature. NEPCM slurry and more conductive materials combined with heat sinks lessen the influence of thermal and frictional entropy creation as well.

Nano-encapsulated phase change material (NEPCM) is compact, has a high specific surface area, is thermally reliable, and has a wide range of potential applications. However, most of the process conditions in use today are rather complex, making it challenging to create NEPCM with good microscopic morphology and outstanding thermal characteristics [25,26,27,28,29]. Liu et al. [30] created a series of NEPCM using a simple sol-gel technique, using disodium hydrogen phosphate dodecahydrate for the core material and silicon dioxide for the shell material. The encapsulation ratio and melting enthalpy of the produced NEPCM reached maximum values of 70.1% and 165.6 J/g, respectively. According to the data, the adequate component ratio and suitable reaction conditions contribute to NEPCM’s superior microscopic morphology and thermal characteristics. Stearic acid (SA)/Ag nanocapsules were synthesized by Huanmei Yuan et al. [31] utilizing a Pickering emulsifier and a chemical reduction process. The results demonstrated that the thermal dependability of the nanocapsules was assessed after 2000 thermal cycles, during which time their latent heat marginally decreased by 0.55%. The fabrication of N-Hexacosane-encapsulated Titania phase change composite using a sol-gel approach was achieved by Khanna et al. [32]. The findings showed that the NEPCM was solidified and liquefied at 52.08 °C and 54.02 °C, respectively, with latent heats of 127.37 J/g and 142.09 J/g. The thermogravimetric curves showed that the composite’s overall thermal stability increased with the increasing titanium concentration. In another study [33], by using a chemical technique, they established how to manufacture silica NEPCM layered between exfoliated-graphite nanosheets. The results indicated that there were no chemical processes that occurring in the phase transition material, which had a diameter of 120–220 nm. Furthermore, at 57.9 °C and 48.1 °C, respectively, with latent heats of 126.7 J/g and 117.6 J/g, the solid–liquid phase transition of the NEPCM nanocomposite was observed. After 300 heat cycles, the NEPCM composites showed very high durability against thermal deterioration and 15.74 W/m K thermal conductivity.

Lately, the suspension of NEPCMs as a novel type of nanofluid was examined in various enclosures. Cao et al. [34] investigated the free convective of NEPCM nanofluid within an insulated chamber with two pipes acting as cooler and heater sources with a constant temperature boundary condition. They demonstrated how the NEPCM phase transition happens at low Rayleigh numbers but that it has no bearing on the heat transmission rate. Instead, it is heavily linked to the thermal conductivity of the nanofluid. The free convective heat transport in NEPCM nanofluid within a square enclosure that has been differentially heated and rotated with a constant, uniform counterclockwise rotational velocity was studied by Alhashash et al. [35]. The rotation parameter primarily influences the number of cell circulations, the number of inner vortexes, and the strength of those vortexes. Higher rotational speed results in less NEPCM phase transition and slower heat transfer. In an angled L-shaped chamber, Sadeghi et al. [36] studied the free convection and entropy formation of NEPCM. The findings show that the micro-rotation parameter, Stefan number, and nondimensional fusion temperature all negatively affected the NC heat transfer of NEPCMs and decrease the Nuavg by up to 42%, 25%, and 15%, respectively. On the other hand, the Nuavg was increased by up to 36% when more nanoparticles were present. Zidan et al. [37] investigated the NEPCM–water mixture NC flow in a reversed T-shaped porous cavity with two heated corrugated baffles. This research shows that increasing the Raleigh number causes escalation velocity fields and phase change zone structural changes, whereas decreasing the Darcy number has the opposite impact. Hussain et al. [38] looked into the free convection of NEPCM in a grooved enclosure with an oval form and saturated with a porous medium. According to the study’s findings, raising the Darcy parameter reduces the porous flow’s resistance, which in turn enhances the streamline strength and nanofluid movement. Furthermore, the enlarged radius of the inner oval form creates a barrier in the grooved cavity, which slows the passage of the nanofluid within.

In the literature, there are no studies on the natural convection of NEPCM in an inversed T-shaped cavity, including a trapezoidal fin subjected to a magnetic field. A better understanding of the impact of the studied parameters on heat transfer rates would be beneficial for design engineers, as cavities saturated with porous media are widely founded in engineering applications, including (but not limited to) heat exchangers and electrical components. In this work, the natural convection of NEPCM in an enclosure saturated with porous media is handled by the high-order GFEM. The run simulations look at how viral parameters affect the contours of temperature, heat capacity ratio, and nanofluid velocity within an inverse T-shaped cavity. These parameters include the Darcy number, the concentration of NEPCM nanoparticles in the base fluid, the Rayleigh number, and the Hartmann number representing the intensity of the magnetic field.

## 2. Problem Formulation

**Figure 1:**
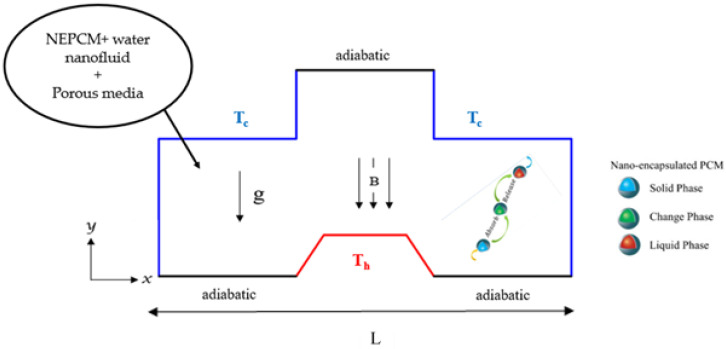
Physical problem.

As shown in Figure 1, we consider an inverted T-shaped porous cavity with a trapezoidal fin at the bottom. The porous cavity is loaded with a nanofluid consisting of water as a base fluid and nano-encapsulated PCM (NEPCM) as nanoparticles. This novel substance’s nanoparticles are made up of an outer shell and an inner core. The core is made of nonadecane, while the exterior is mostly made of polyurethane. Their thermal properties are shown in Table 1. The fusion temperature, which is restricted to this range $T_{h}<T_{f}<T_{c}$ is what characterizes the single-particle core. The latent heat of the core and the phase transition temperatures are estimated to be 211 (kJ/kg) and 305 (K), respectively. Overall, the PCM cores’ capacity to absorb, store, and release heat energy distinguishes them. When combined with a base liquid such as water, they also effectively transmit heat energy.

The governing equations are as follows [39,40]:

The side walls are maintained at low temperature, *T_c_*, while the trapezoidal fin walls are heated and kept at high temperature, Th with (*T_h_* > *T_c_*), and the other walls are thermally isolated. For this problem, the flow is assumed to be steady and laminar. The driving force in the geometry under study is the buoyancy force due to the temperature difference between the trapezoidal fin and the sidewalls. A uniform magnetic field is applied. It is believed that the effects of Joule heating, radiation, displacement currents, and viscous dissipation are insignificant. Natural convection is approximated using the Boussinesq approximation in the buoyancy element of the momentum equation. Pressure adjustments do not affect the density of nanoliquids. Temperature gradients, on the other hand, alter the density. The particles are distributed uniformly throughout the host fluid, and dynamic and thermal equilibrium between the nano-additives and the base fluid is established.

(1) ∇⋅v=0

(2) $$\rho_{b}\cdot \nabla v=-\nabla p+\nabla \cdot \left(\mu_{b}\nabla v\right)+{\left(\rho \beta \right)}_{b}g\left(T-T_{c}\right)-\frac{\sigma_{nf}B_{0}^{2}v}{\rho_{nf}}$$

(3) $${\left(\rho C_{p}\right)}_{b}v\cdot \nabla T=\nabla \cdot \left(k_{b}\nabla T\right)$$

The current suspension is distinguished by its global density, which is expressed as [41]

(4) $$\rho_{b}=\left(1-\phi \right)\rho_{f}+\phi \rho_{p}$$

where the symbols f, and p denote the base fluid and the added nanoparticles, respectively.

The nanoparticle density of NePCM is provided below:

(5) $$\rho_{p}=\frac{\left(1+t\right)\rho_{\mathrm{co}}\rho_{\mathrm{sh}}}{\rho_{\mathrm{sh}}+i\rho_{\mathrm{co}}}$$

where the symbols $\rho_{\mathrm{sh}\;}$, $\rho_{\mathrm{co}}$ and ι denote the shell’s density, the core’s density and the mass ratio of the core-shell (ι∼0.447) [41], respectively.

Additionally, the core density of a PCM is the average of its solid and liquid phases. When using NEPCM, the water’s specific heat capacitance value may be calculated as

(6) $$C_{p,b}=\frac{\left(1-\phi \right){\left(\rho C_{p}\right)}_{f}+\phi {\left(\rho C_{p}\right)}_{p}}{\rho_{p}}$$

Heat capacitance is considered for a single-phase state, *ip*, *p*, is defined according to the following expression:

(7) $$C_{p,p}=\frac{\left(C_{p,co}+\iota C_{p,sh}\right)\rho_{\mathrm{co}}\rho_{\mathrm{sh}}}{\left(\rho_{\mathrm{sh}}+\iota \rho_{\mathrm{co}}\right)\rho_{p}}$$

The heat capacity of the inner substance (core) is considered the average of the heat capacities of both states, solid and fluid. This is because whether the nanoparticle’s core is in a solid or fluid state, the latent heat is changed in the form of the heat capacity of the NEPCM. This new form of heat can be defined as follows [42]:

(8) $$C_{P,p}=C_{P,co}+\frac{h_{sf}}{T_{Mr}}$$

(9) $$C_{P,P}=C_{p,:00}+\left\{\frac{\pi }{2}\cdot \left(\frac{h_{sf}}{T_{Mr}}-C_{P,c0}\right)\cdot \sin\left(\pi \frac{T-T_{fu}+\left(T_{Mr}/2\right)}{T_{Mr}}\right)\right\}$$

(10) $$C_{P,P}=C_{p,c0}+2\left(\frac{h_{fs}}{T_{Mr}^{2}}-\frac{C_{p,C0}}{T_{Mr}}\right)\left(T-T_{fu}+\frac{T_{Mr}}{2}\right).$$

Most of the researchers employ linear interpolation to deal with the phase change due to it is simplicity. However, in this work, we chose to characterize phase change by employing the sine function to assure function continuity in the whole domain, where $T_{Mr}$ is the range of the temperature. This interval circumvents the discontinuity in the stability of energy. The total heat capacity of the NEPCM core incorporating fusion temperature and the sensible is determined based on $T_{Mr^{*}}$

(11) $$C_{p,p}=C_{p,co}+\left\{\frac{\pi }{2}\cdot \left(\frac{h_{sf}}{T_{Mr}}-C_{p,c0}\right)\cdot \sin\left(\pi \frac{T-T_{fu}+\left(T_{Mr}/2\right)}{T_{Mr}}\right)\right\}\gamma$$

where

(12) $$\gamma =\{\begin{array}{c} 0,T<T_{fu}-\frac{T_{Mr}}{2}\\ 1,T_{fu}-\frac{T_{Mr}}{2}<T<T_{fu}+\frac{T_{Mr}}{2}\\ 0,T>T_{fu}+\frac{T_{Mr}}{2} \end{array}$$

The suspension’s thermal volume expansion rate is calculated as

(13) $$\beta_{b}=\left(1-\phi \right)\beta_{f}+\phi \beta_{p^{-}}$$

To estimate the thermal conductivity of a combination including nano-encapsulated particles, the following definitions are stated [43]:

(14) $$\frac{k_{b}}{k_{f}}=1+Nc\phi .$$

The dynamic conductivity of suspension is

(15) $$\frac{\mu_{b}}{\mu_{f}}=1+Nv\phi$$

where Nc  and Nv  in the above expressions define the numbers of thermal conductivity and viscosity, respectively.

The higher the thermal conductivity and viscosity values, the higher the increment in the mixture’s thermal conductivity and dynamic viscosity (water and PCM particles). These constant values were established by Ghalambaz et al. [44] for several hybrid nanofluids and nanofluids. It is determined that these expressions are acceptable only for nanofluids if ϕ<5%. The used quantities were considered in dimensionless form as follows:

$$\begin{array}{ll} & X=\frac{x}{L^{\prime }},\\ & Y=\frac{y}{L^{\prime }}\\ & \delta =\frac{\delta^{*}}{L}\\ & U=\frac{uL}{\alpha_{f}}\\ & V=\frac{vL}{a_{f}}\\ & P=\frac{p\ell^{2}}{\rho_{f}\alpha_{f}^{2}}\\ & \Theta =\frac{T-T_{c}}{T_{h}-T_{c}} \end{array}$$

The nondimensional mathematical formulations become

(16) $$\frac{\partial U}{\partial X}+\frac{\partial V}{\partial Y}=0$$

(17) $$\left(\frac{p_{b}}{\rho_{f}}\right)\left(U\frac{\partial U}{\partial X}+V\frac{\partial U}{\partial Y}\right)=\frac{\partial P}{\partial X}+\mathrm{Pr}\left(\frac{\mu_{b}}{\mu_{f}}\right)\left(\frac{\partial^{2}U}{\partial X^{2}}+\frac{\partial^{2}U}{\partial Y^{2}}\right)$$

(18) $$\left(\frac{p_{t}}{\rho_{f}}\right)\left(U\frac{\partial V}{\partial X}+V\frac{\partial V}{\partial Y}\right)=\frac{\partial P}{\partial Y}+\mathrm{Pr}\left(\frac{\mu_{b}}{\mu_{f}}\right)\left(\frac{\partial^{2}}{\partial X^{2}}+\mathrm{RaPr}\frac{{\left(\rho \beta \right)}_{b}}{{\left(\rho \beta \right)}_{f}}\right)\Theta$$

(19) $$\mathrm{Cr}\left(U\frac{\partial \Theta }{\partial X}+V\frac{\partial \Theta }{\partial Y}\right)=\frac{k_{b}}{k_{f}}\left(\frac{\partial^{2}\Theta }{\partial X^{2}}+\frac{\partial^{2}\Theta }{\partial Y^{2}}\right)-\frac{\sigma_{hnf}}{\sigma }Ha^{2}V$$

The dimensionless boundary conditions are

$$\begin{array}{ll} & U=V=0,\Theta =1\;\mathrm{on}\;\mathrm{the}\;\mathrm{trapezoidal}\;\mathrm{fin}\;\mathrm{at}\;\mathrm{the}\;\mathrm{bottom}\\ & U=V=0,\Theta =0\;\mathrm{on}\;\mathrm{the}\;\mathrm{side}\;\mathrm{walls}\\ & U=V=0,\frac{\partial \Theta }{\partial Y}=0\;\mathrm{on}\;\mathrm{the}\;\mathrm{rest}\;\mathrm{adiabatic}\;\mathrm{walls}, \end{array}$$

and Ra and Pr are no dimensional quantities of Rayleigh and Prandtl numbers, respectively:

(20) $$\mathrm{Ra}=\frac{g\rho_{f}\beta_{f}\Delta Te^{3}}{\alpha_{f}\mu_{f}}$$

(21) $$\mathrm{Pr}=\frac{\mu_{f}}{\rho_{f}\alpha_{f}}$$

also,

(22) $$\left(\frac{\rho_{b}}{\rho_{f}}\right)=\left(1-\phi \right)+\phi \left(\frac{\rho_{p}}{\rho_{f}}\right)$$

(23) $$\left(\frac{\beta_{b}}{\beta_{f}}\right)=\left(1-\phi \right)+\phi \left(\frac{\beta_{p}}{\beta_{f}}\right)$$

Given that it is presumed that the thermal expansion of water is equivalent to that of NEPCMs, $\left(\beta_{b}/\beta_{f}\right)∼1$, Cr describes the ratio of heat capacity of the suspension over the water heat capacity: $Cr=\frac{{\left(\rho C_{p}\right)}_{b}}{{\left(\rho C_{p}\right)}_{f}}=\left(1-\phi \right)+\phi \lambda +\frac{\phi }{\delta \;\mathrm{Ste}\;}f.\;$ (Ste) is the Stefan number, and it is defined as follows:

(24) $$\lambda =\frac{\left(C_{p,co}+uC_{p,sh}\right)\rho_{cof}\rho_{\mathrm{sh}}}{{\left(\rho C_{p}\right)}_{f}\left(\rho_{\mathrm{sh}}+\rho_{\mathrm{co}}\right)}$$

(25) $$\varepsilon =\frac{T_{Mr}}{\Delta T}$$

(26) $$\mathrm{Ste}=\frac{{\left(\rho C_{p}\right)}_{f}\Delta T\left(\rho_{\mathrm{sh}}+\rho_{\mathrm{co}}\right)}{\alpha_{f}\left(h_{sf}\rho_{\mathrm{co}}\rho_{\mathrm{sh}}\right)}$$

Additionally, the nondimensional fusion expression, f, is given as

(27) $$f=\frac{\pi }{2}\sin\left(\frac{\pi }{\varepsilon }\left(\Theta -\Theta_{fu}+\frac{\varepsilon }{2}\right)\right)\sigma$$

where

(28) $$\sigma =\{\begin{array}{ll} 0, & \Theta <\Theta_{\mathrm{fu}}-\frac{\varepsilon }{2},\\ 1, & \Theta_{\mathrm{fu}}-\frac{\varepsilon }{2}<\Theta <\Theta_{\mathrm{fu}}+\frac{\varepsilon }{2}\\ 0, & \Theta >\Theta_{\mathrm{fu}}+\frac{\varepsilon }{2}. \end{array}$$

Here, $\Theta_{fu}$, the nondimensional fusion temperature is

(29) $$\Theta_{fu}=\frac{T_{fu}-T_{c}}{\varepsilon T}.$$

The local Nusselt number of the heated side is obtained:

(30) $$Nu=-\left(1+Nc\phi \right)\frac{\partial \Theta }{\partial Y}$$

Additionally, the averaged form of the Nusselt number of the heated side is given as

(31) $$\bar{Nu}=\int_{-0.5}^{0.5}NudY$$

## 3. Numerical Methodology

Various numerical methods can be exploited in order to find solutions of the differential equations whether in parallel or in sequential (see, e.g., [45,46,47,48]). Here, the main equations and accompanying initial boundary conditions are addressed using the Galerkin finite element method. The non-linear partial differential equations are transformed into linear equations using the weighted residual technique [42]. 

For the validation, the (Nu) on the hot surface at (Re = 500, Ha = 0, ϕ = 4% and N = 4) is utilized. Table 2 displays the results of the mesh independence study. The findings illustrate that the grid size of 21,999 is the ideal option.

**Figure 2:**
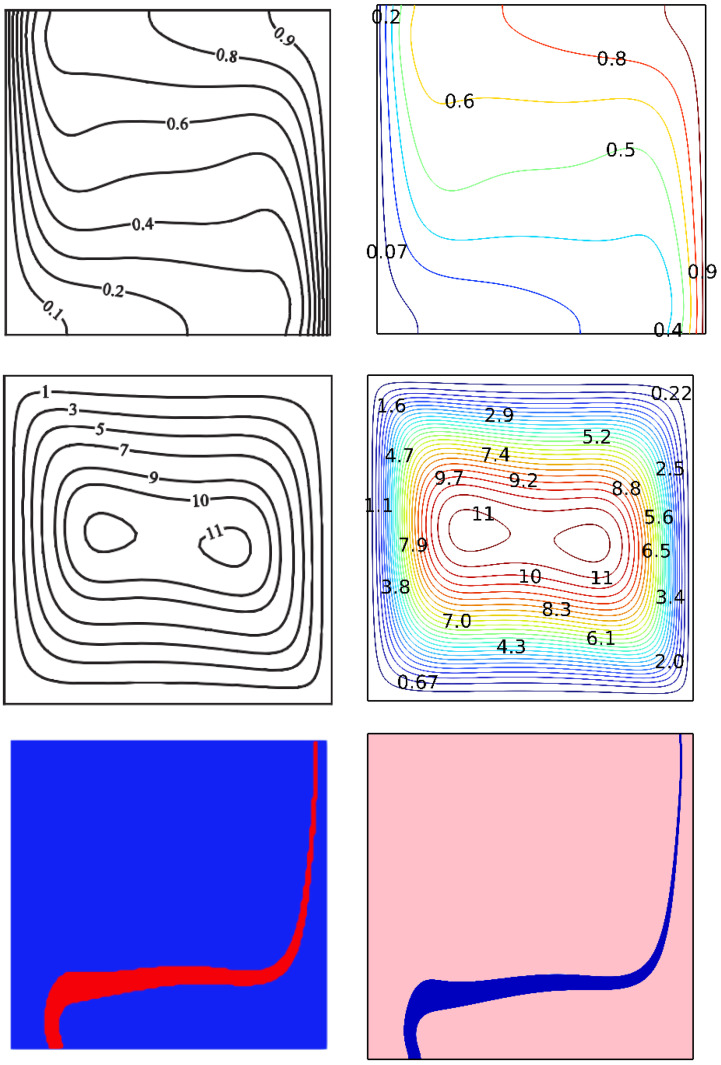
Comparison of current work with that of Mohammad Ghalambaz et al. reprinted/adapted with permission from Ref. [44]. 2022, Elsevier.

By using numerical research, the existing results were validated. The results from the model used in the current study are compared to those presented in Ghalambaz et al. [44], as seen in Figure 2.

## 4. Results and Discussion

In this important part of the research, we present the results that show the impacts of Darcy’s number (Da), Hartmann’s number (Ha), Rayleigh’s number (Ra) and the concentration of PCM nanoparticles on thermal activity and how the suspension (PCM nanoparticles + water) moves inside the room. 

We note a scientific fact that occurs in a fluid: when the fluid absorbs a quantity of thermal energy, its weight decreases and, therefore, begins to move upward as it shifts downward. In contrast, the fluid moves downward as it loses heat energy. This kind of heat transfer accompanied by mass transfer is called Buoyancy-driven flow. 

**Figure 3:**
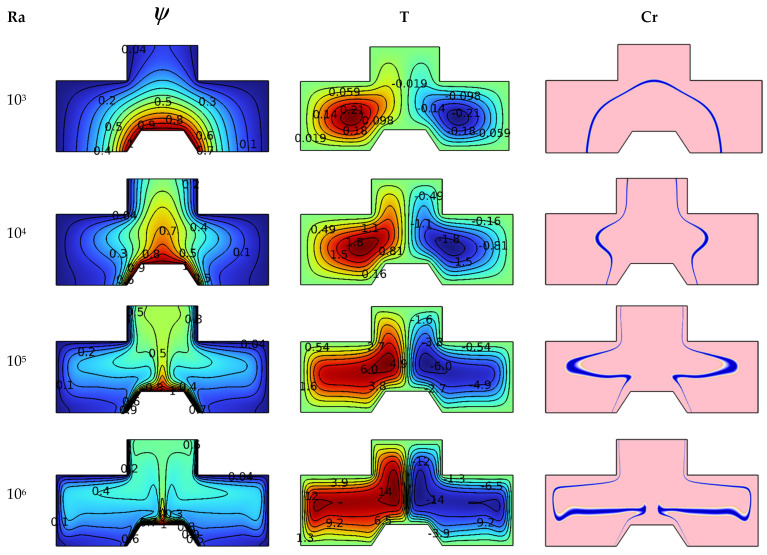
Ra number influence on streamlines, isotherms, and Cr for Da = 10^−2^, Ha = 0, and φ = 4%.

Figure 3 is devoted to understanding the effect of the Ra (between 10^3^ and 10^6^) number on the studied system for Ha = 0, Da = 10^−2^ and φ = 4%. This understanding is achieved by analyzing the contours of isotherms (dimensionless temperature), streamlines (line paths) and phase-changing zone (heat capacity Cr). The dimensionless temperature (isotherms) shows a gradual temperature distribution from the hot fin toward the cold walls of the container. We note that the fluid layers adjacent to the fin have a high temperature. Then this distribution is centered over the fin in an upward direction. In contrast, cold fluid layers are centered near the lateral walls. It is noted that the intensity of this distribution increases in terms of the number Ra. The streamlines are exactly equivalent to the isotherm patterns, meaning that the fluid layers above the trapezoidal fin move toward the top, while the cold layers on the lateral edges of the chamber move downwards. Finally, we note the formation of a circular motion of the suspension flow. This movement is divided into symmetric parts, one on the right side with the same clockwise direction and the second on the left side in the anticlockwise direction. We also note that the intensity of the movement of the two vortices increases in terms of the number Ra, whereas the center of this recirculation zone moves upward as the value of Ra increases.

When Ra = 10^6^, the intensity of thermal buoyancy becomes very high, and we notice that the center of the vortex is divided into two parts, the first near the cold wall and the second near the hot fin. For the heat capacity contours (Cr), we notice an effect of the number Ra on the heat capacity (Cr). We notice that there is a line in the form of a semicircle around the trapezoidal fin for Ra = 10^3^, then it is divided into two symmetric parts as the number Ra increases. The blue lines indicate the regions where the change of physical state occurs for PCM nanoparticles. We notice that the lines expand in terms of Ra because the movement of suspension particles increases in terms of Ra. In addition to this, it is noticed that the two heat capacity lines are thinner, close to both cold and hot walls, while they are thicker in the middle of the room. The greater the temperature gradient, the thinner the thickness of the equivalent heat capacity line for the zones where the change of physical state occurs. 

**Figure 4:**
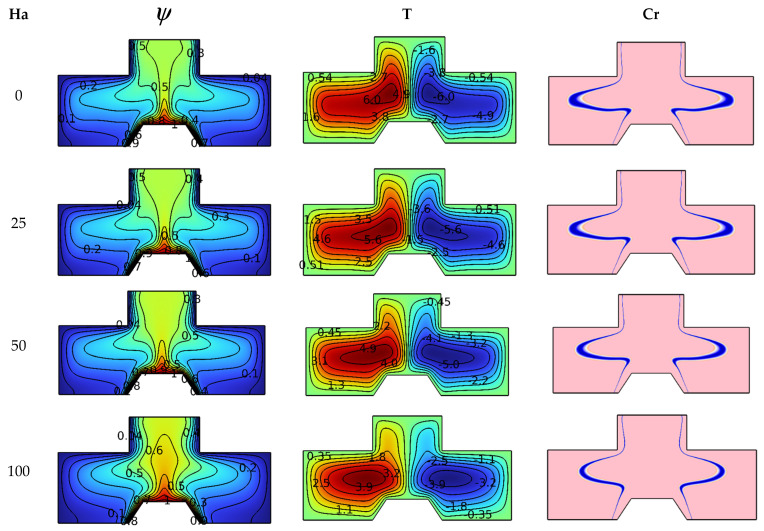
Ha number influence on streamlines, isotherms, and Cr for Ra = 10^5^, Da = 10^−2^, and φ = 4%.

In order to understand the impact of the values of Hartmann number (between the values 0 to 100) on the studied system, Figure 4 illustrates this effect of Da = 10^−2^, Ra = 10^5^ and φ = 4%. The scientific explanation for the influences of the Hartmann number is found by analyzing the contours of isotherms, streamlines and heat capacity (Cr). It is known that the magnetic field creates an electromagnetic force (Lorentz force) that hinders the movement of fluid particles, so we notice a gradual decline in isotherm distribution in terms of the number Ha. The streamlines also show a decrease in the intensity of vorticity by increasing the value of Ha. The contours of heat capacity (Cr) show that the blue lines of physical state change converge as the value of Ha increases because the velocity of the suspension particles decreases in terms of Ha.

**Figure 5:**
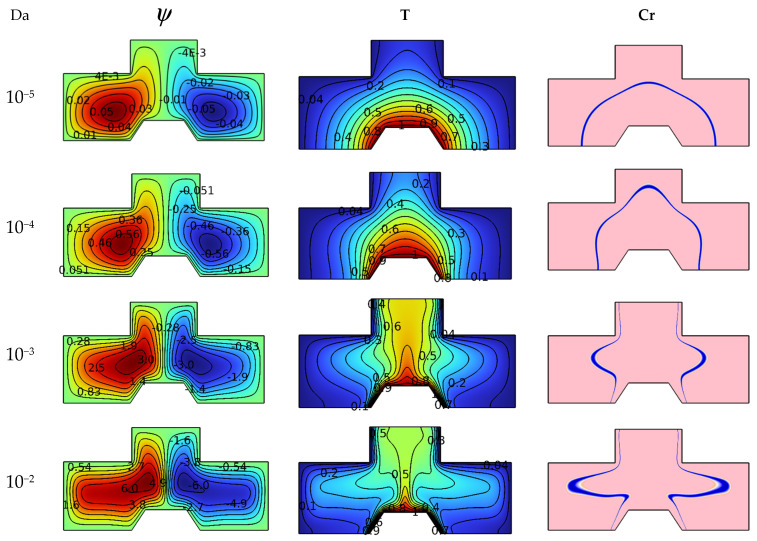
Da number influence on streamlines, isotherms, and Cr for Ra = 10^5^, Ha = 0, and φ = 4%.

The results of this work also include the effect of the medium permeability on the studied system, so Figure 5 illustrates the influences of the Darcy number (between Da = 10^−2^ and Da = 10^−2^) on each of the isotherms, heat capacity and streamlines for Ra = 10^5^, Ha = 0, and φ = 4%. It is known that the permeability of the space is defined in terms of the Darcy number; that is, the higher the value of this number, the greater the permeability. Based on this, the isotherms show an expansion of the dimensionless temperature in terms of Darcy’s number because the suspension movement becomes easier. On the other hand, the streamlines depict an increase in the intensity of the vortices and an increase in their sizes as the value of Da increases. Heat capacity contours are also influenced by the Darcy number because the increase in the speed of the flow expands the blue lines of the regions with the change in the physical state of nano-encapsulated PCM. 

**Figure 6:**
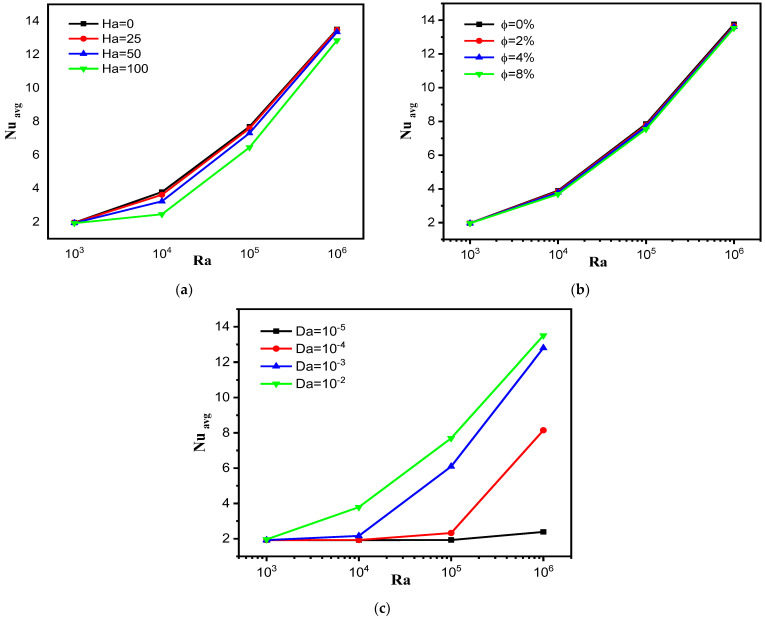
The effect of (**a**) Ha number, (**b**) nanoparticle volume fraction and (**c**) Da number on the Nu _avg_ for different values of Ra.

Figure 6 presents the developments of the mean values of the Nusselt number (Nu) of the hot trapezoidal fin in terms of Da, Ha, Ra and φ. Figure 6a is intended to show the effect of numbers Ha and Ra on Nu. For φ = 4% and Da = 10^−2^ we notice that the values of Nu increase with increasing values of Ra and decrease as the values of Ha increase, just as expected. That is, the higher the speed of the suspension particles, the better the convective heat transfer, and accordingly, the values of the number Nu increase. In addition to this, we notice that the increase in the values of Nu in terms of Ra gradually changes with the increase in the value of Ha. That is, as the value of Ha increases from 0 to 100, the evolution becomes nonlinear. Figure 6b is intended to present the influence of the nano-encapsulated PCM concentration and Ra on Nu. For Ha = 0 and Da = 10^−2^ we notice that in this studied space, there is no strong influence of the nano-encapsulated PCM concentration on Nu. The negligible effect of the ratio of NEPCM on Nu can be explained by the finite motion of the flow within the chamber. Figure 6c presents the effect of the Darcy number, i.e., the porosity of the medium and Ra number on Nu of the hot surfaces of trapezoidal fin for Ha = 0 and φ = 4%. We note an effective effect of the medium permeability (Da number) on Nu values. That is, the values of Nu increase with augmenting Da. The latter can be explained by the following: the expansion of the medium’s permeability means a decrease in the flow obstruction, which allows for enhanced convective heat transfer. 

**Figure 7:**
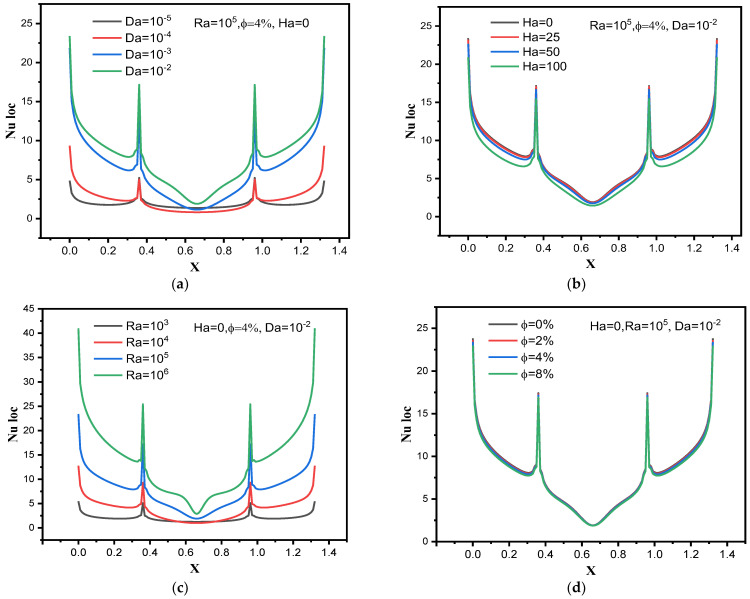
Effects of (**a**) Da number, (**b**) Ha number, (**c**) Ra number and (**d**) nanoparticle volume fraction on the local Nusselt number.

In order to know the quality of the convective heat transfer along the hot surfaces of trapezoidal fin, Figure 7 presents the distribution of the local values of Nu in terms of Da, Ra, and Ha and φ. Figure 7a illustrates the effect of Da on the local distribution of Nu for Ra = 10^5^, φ = 4% and Ha = 0. We note that the local values of Nu on both sides of the trapezoidal fin are greater than the values in the middle of the fin because the velocity of suspension particles on both sides of the fin is greater. In addition, raising the number Da increases the local values of Nu number along the fin. Figure 7b is intended to display the local distribution of Nu number in terms of Ha for Ra = 10^5^, φ = 4% and Da = 10^−2^. All local values of Nu decrease with increasing Ha along the heated surfaces of the fin. Figure 7c is devoted to presenting the impact of the Ra number on local values of Nu along the heated fin for Ha = 0, φ = 4% and Da = 10^−2^. It is clearly demonstrated that raising Ra increases all local values of Nu along the heated surfaces. Figure 7d is devoted to showing the concentration’s impact on the local Nu distribution for Ha = 0, Ra = 10^5^ and Da = 10^−2^. We note that there is a very small effect of this element on the local values of Nu.

## 5. Conclusions

Through this work, we were able to create a digital simulation for suspension inside a room in the form of an inversed T shape. The suspension consists of water and the elements of nano-encapsulated PCM. The chamber is permeable and has a compound bottom with a hot trapezoidal fin, while the lateral ends are cold. We sought, through the simulation results, to highlight the heat transfer of free convection form between hot and cold elements by using the suspension as a thermal conductor. The study was also carried out under the effect of the intensity of the magnetic field. Analyzing the results of this research, we concluded the following:

- Increasing the porosity of the container makes the flow velocity better, increasing heat transmission.
- Rayleigh number controls the thermal buoyancy force; it was found that increasing this force moves the suspension flow better and has a positive effect on heat transfer.
- The magnetic field creates a force that opposes the movement of the flow. Therefore, the stronger the magnetic field, the lower the flow speed, and consequently, increased thermal activity.
- The movement of the flow within the space is characterized by the development of two identical vortices, as the higher the speed of the flow, the more the development of the vortices is served.
- The bar indicating the location of the change in the physical state of PCM elopements is characterized by two states: the first is the presence of a single band hovering around the heated surface for the low speed of the flow; the second case is characterized by the presence of two opposite bands, one on the right and the other on the left when the flow speed is high.

## Figures and Tables

**Table 1:** Thermophysical properties of the shell and core of the NEPCMs and the base fluid.

Material	$\beta \left(K^{-1}\right)$	C(kJ/kg K)	k(W/m K)	$\rho \left(kg/m^{3}\right)$
Polyurethane: shell	$17.28\times 10^{-5}$	1.3177		786
Nonadecane: core		2.037		721
Water: base fluid	$21\times 10^{-5}$	4.179	0.613	997.1

**Table 2:** Grid independence test for Re = 100, Ha = 0, ϕ = 4% and N = 4.

No. of Elements	1141	2318	5400	21,999	81,359
$\Psi_{\max}$	33.072	33.175	33.236	33.225	33.221
$Nu_{a}$	10.602	10.624	10.622	10.624	10.624

## Data Availability

Not applicable.
